# Supplementary figures and images for: Activity-based probe profiling of RNF12 E3 ubiquitin ligase function in Tonne-Kalscheuer syndrome
Source: Life Sci Alliance. 2022 Jun 28;5(11):e202101248. doi: 10.26508/lsa.202101248 (PMC9240097; doi:10.26508/lsa.202101248)

B

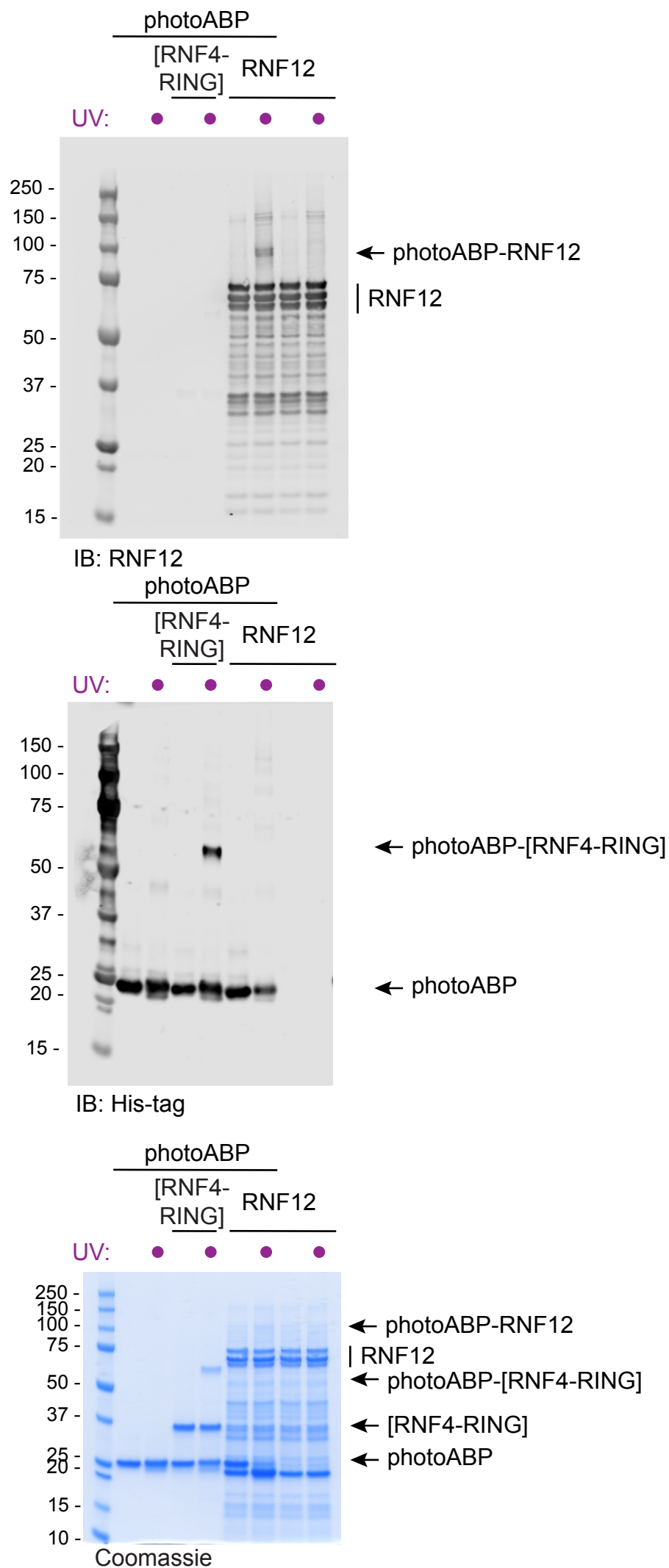

C

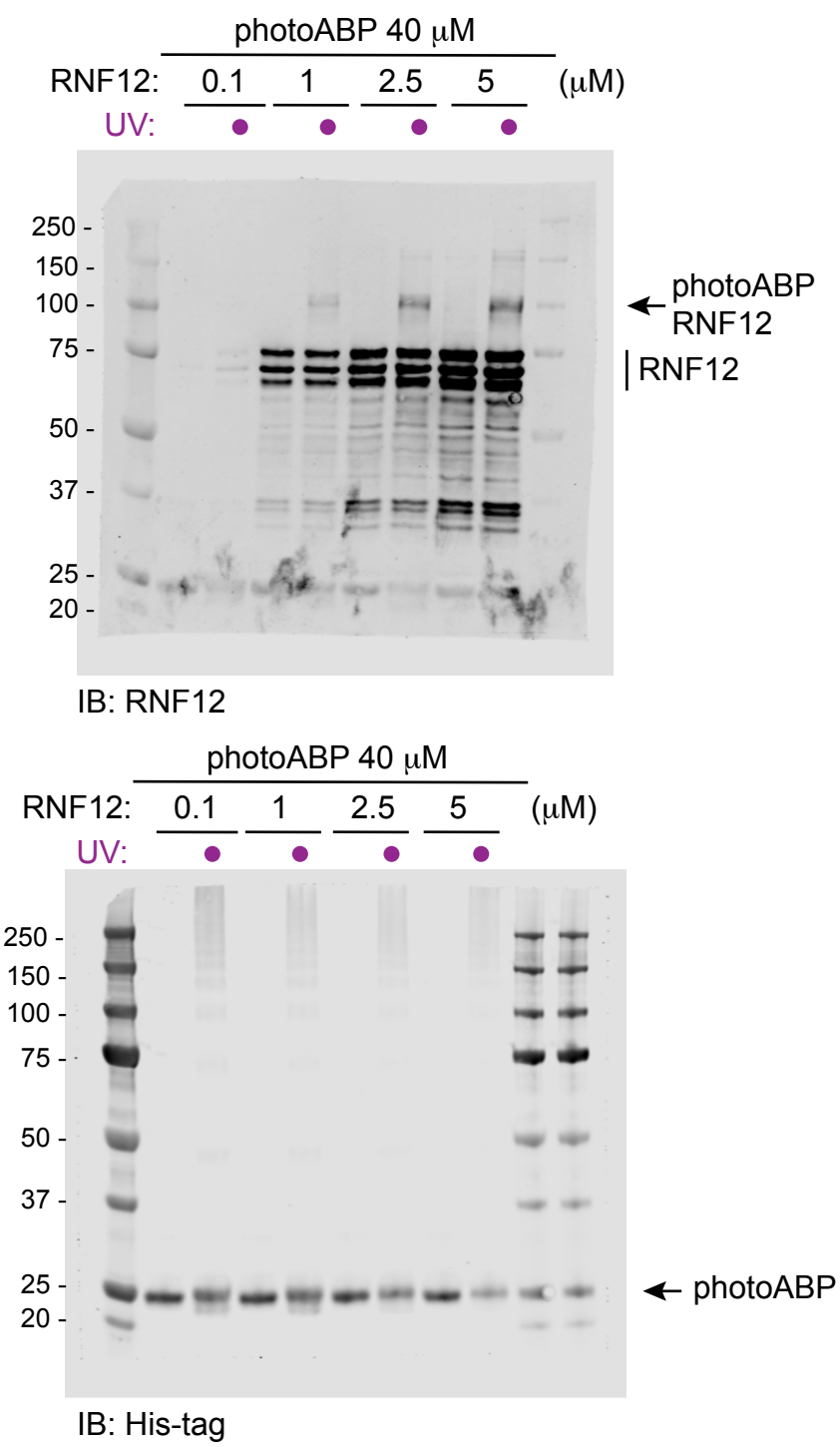

D

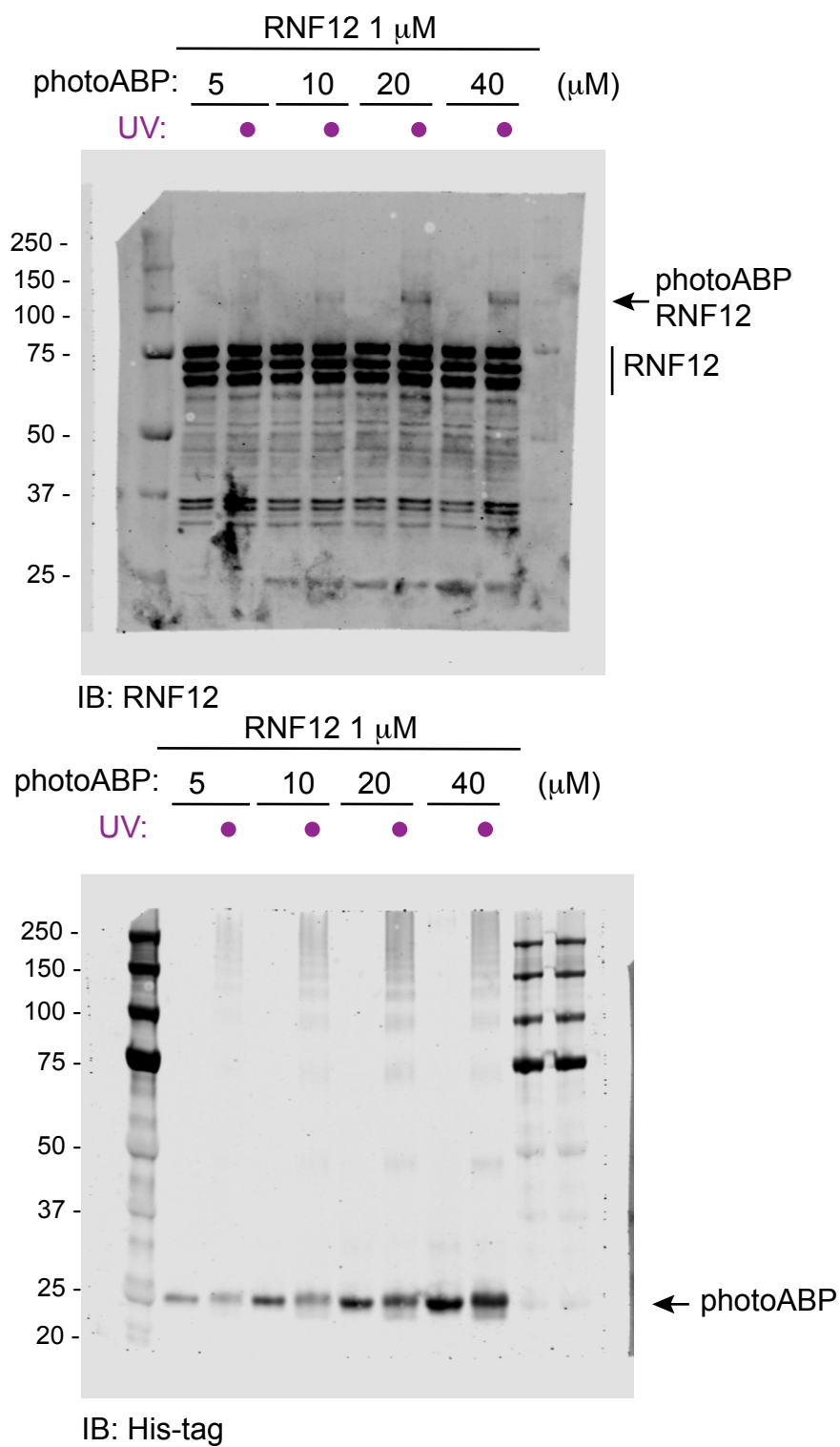

Supplement: Supplementary file 1 [file LSA-2021-01248_SdataF1.pdf]

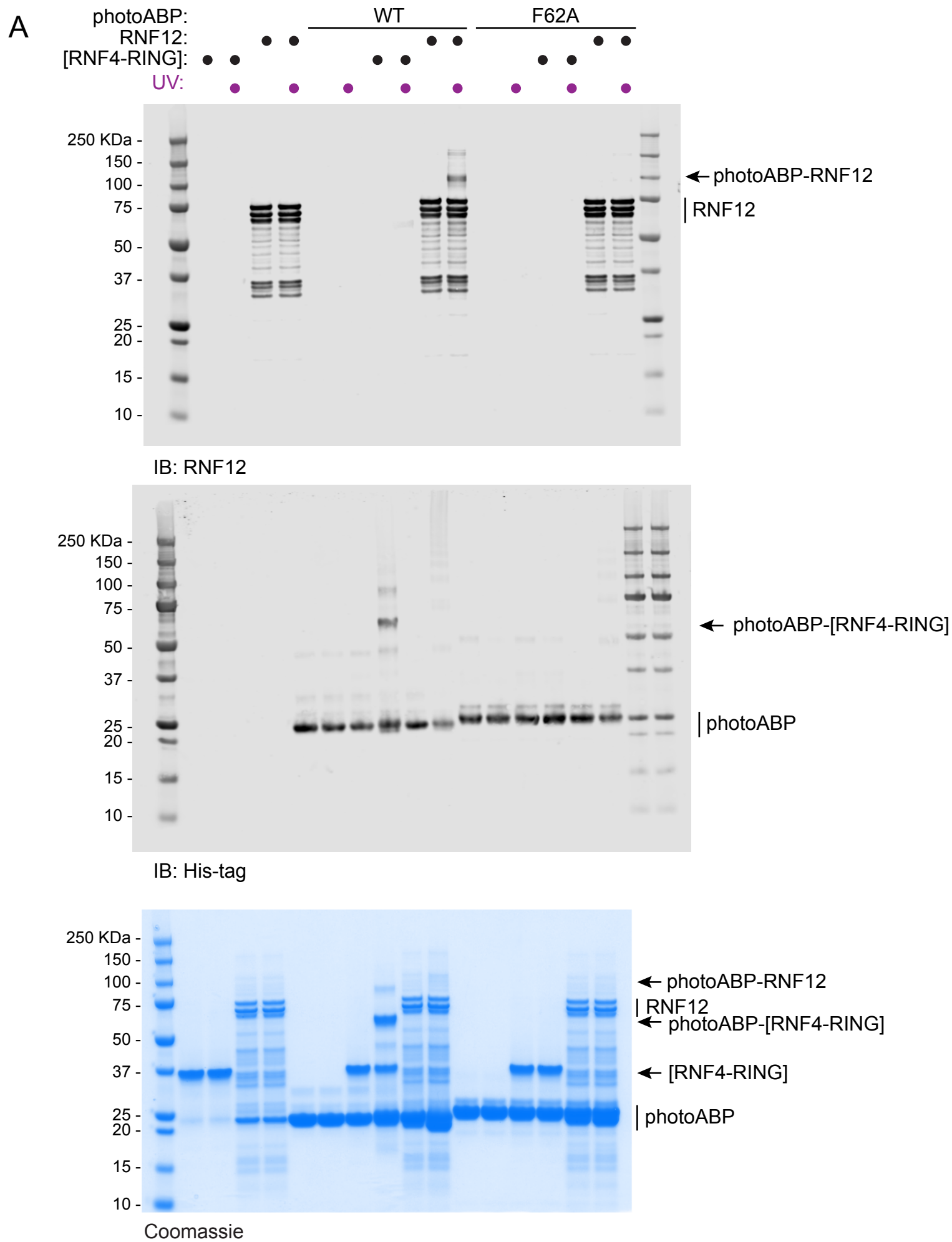

B

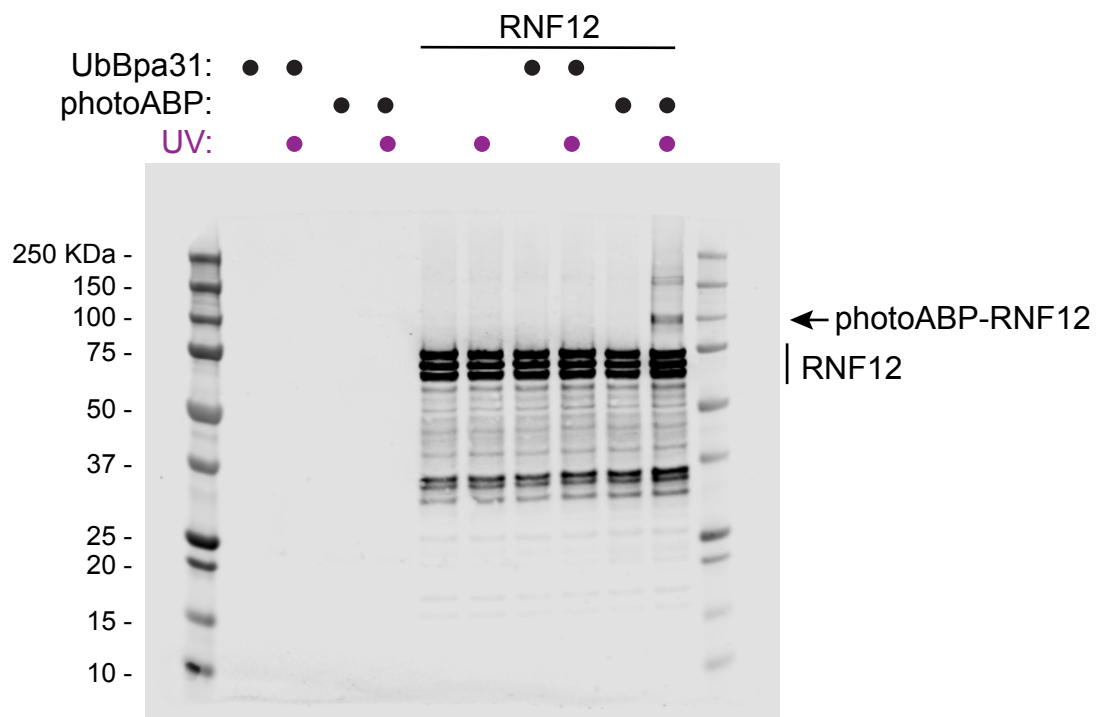

IB: RNF12

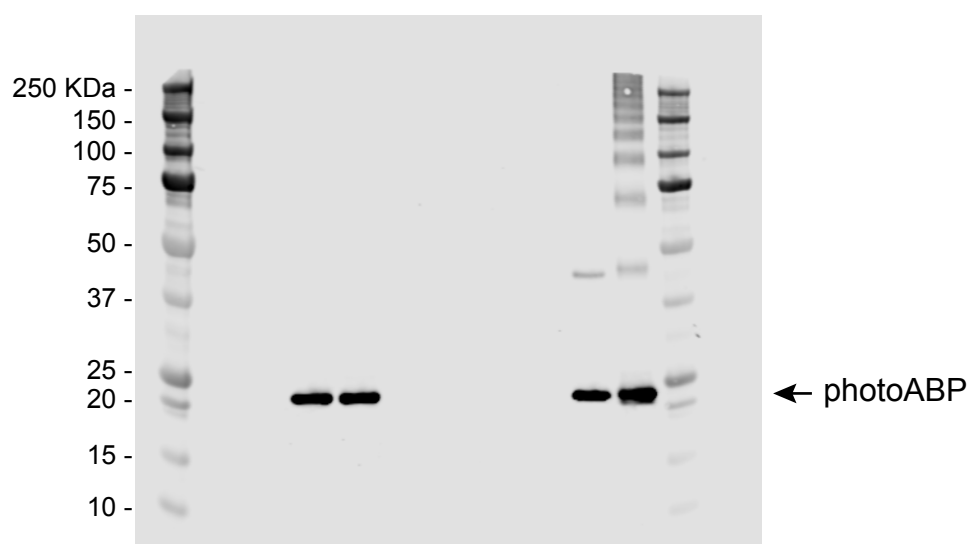

IB: His-tag

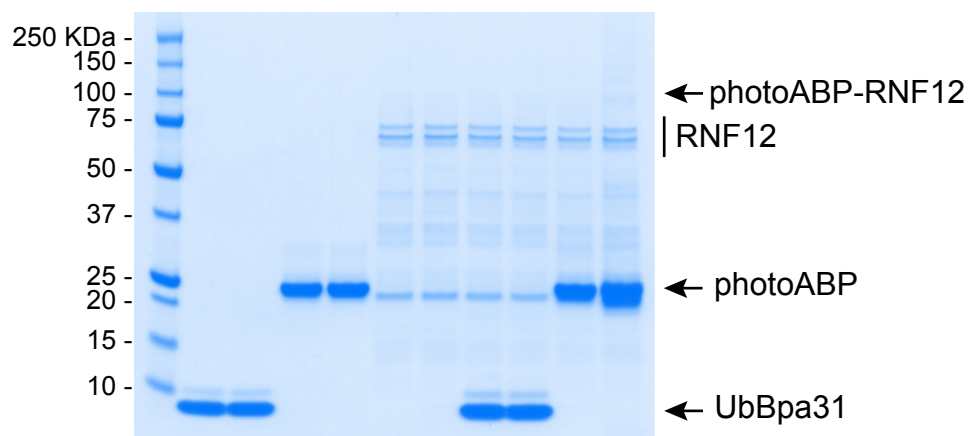

Coomassie

C

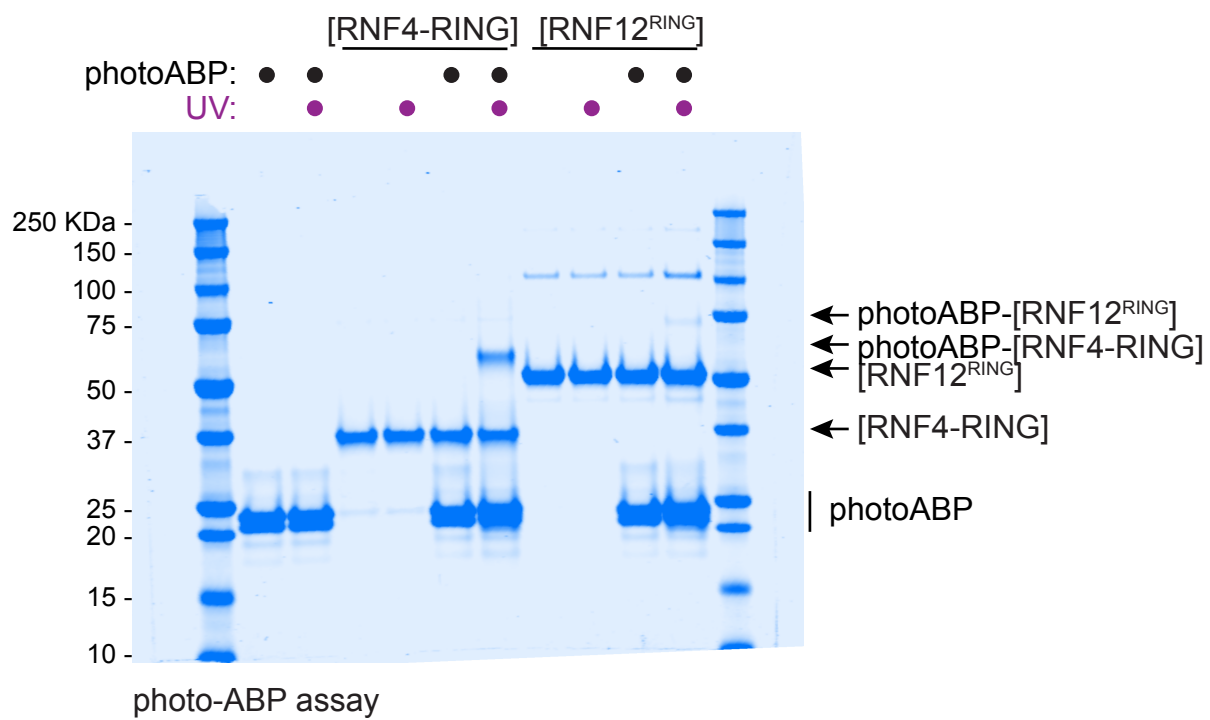

D

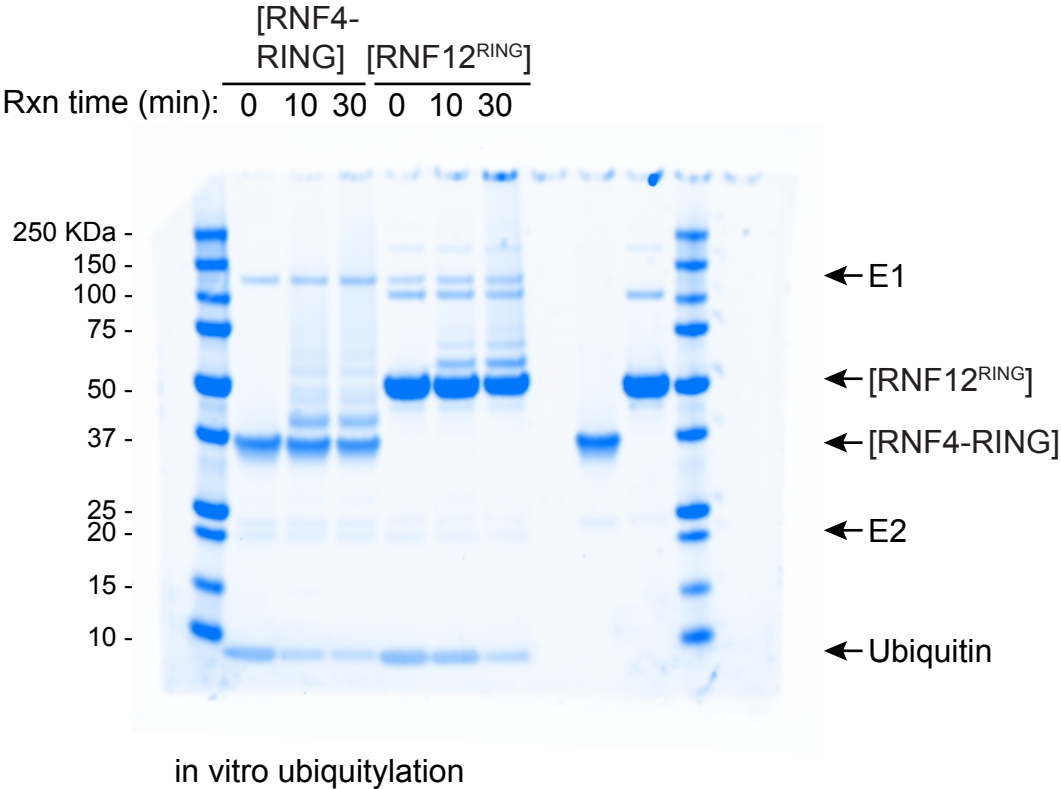

Supplement: Supplementary file 2 [file LSA-2021-01248_SdataF2.pdf]

B

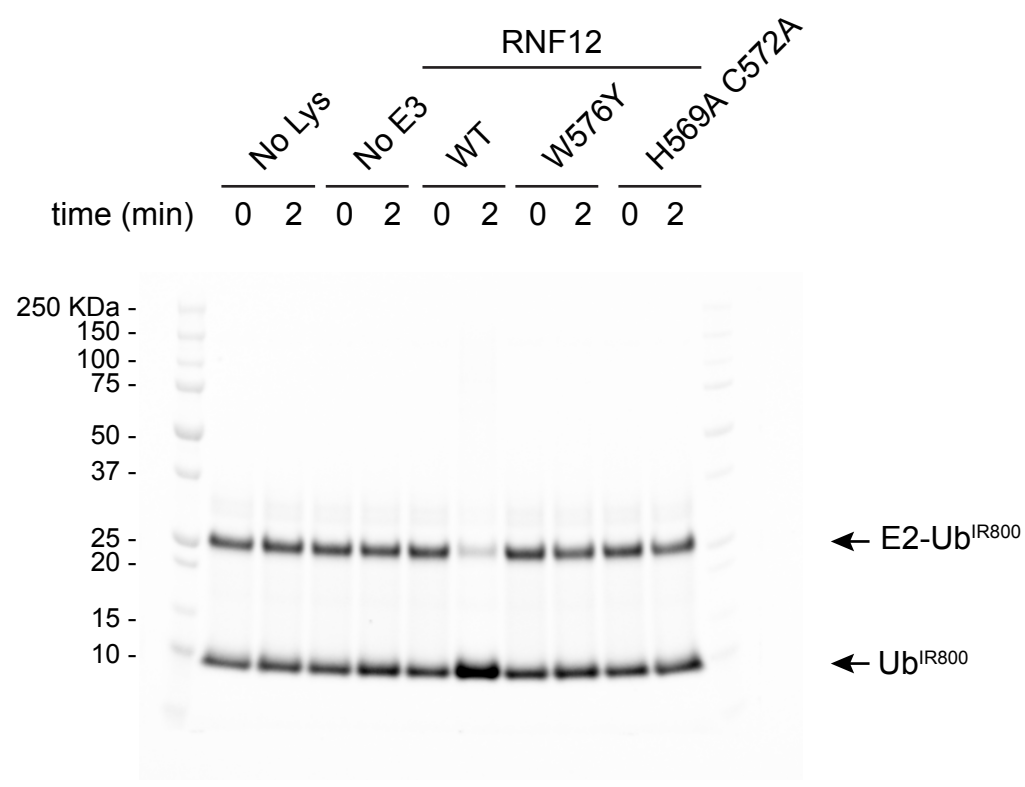

IR Scan

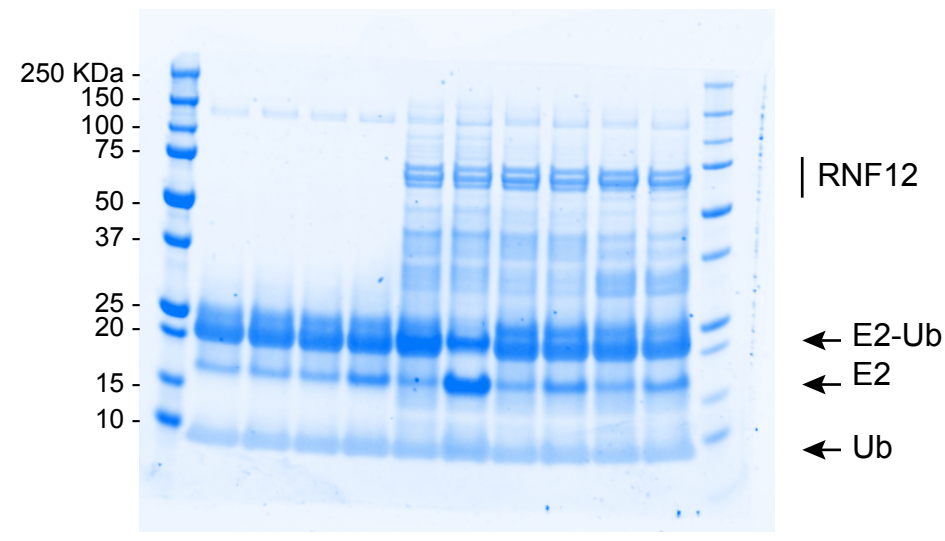

Coomassie

C

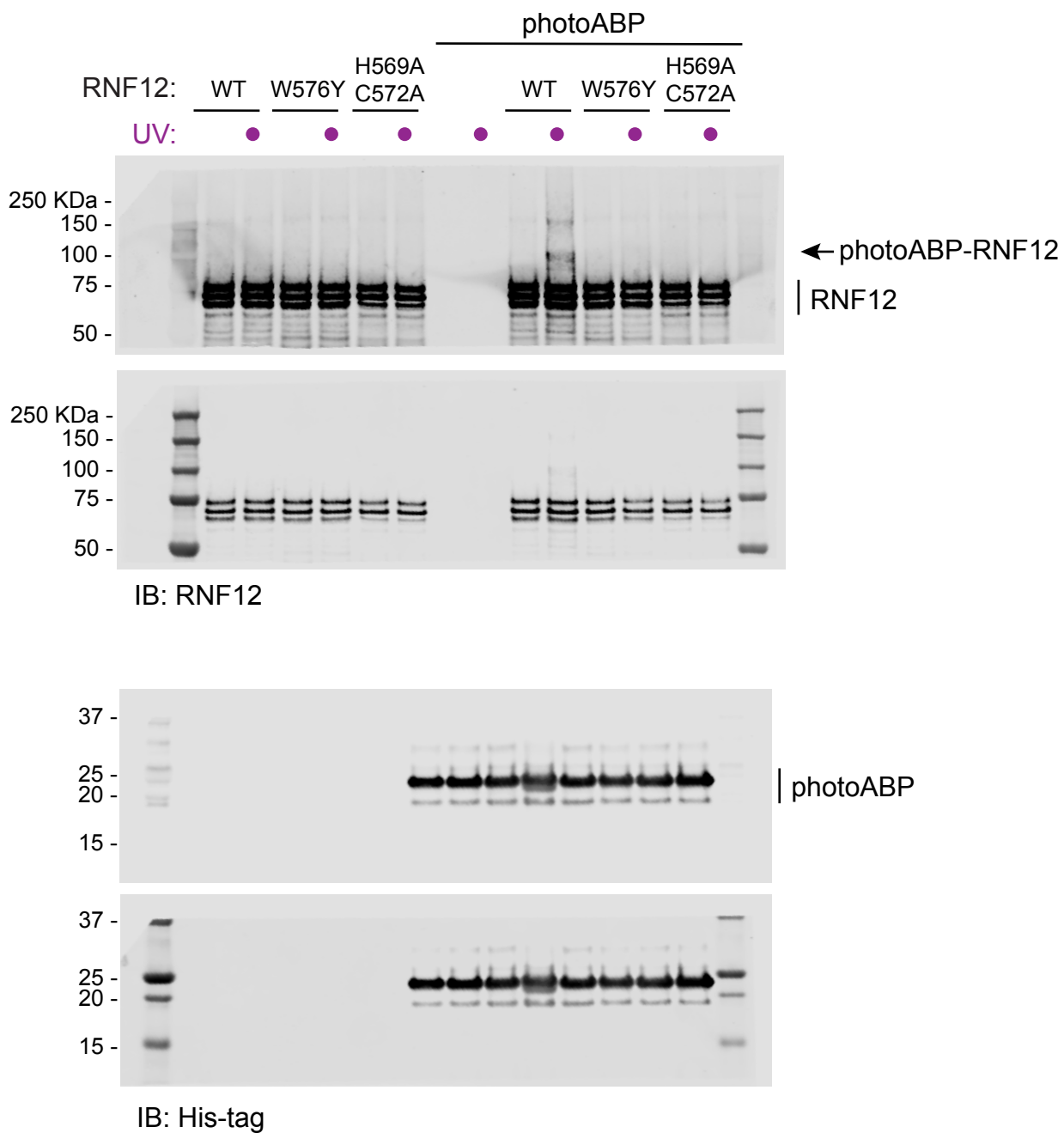

D

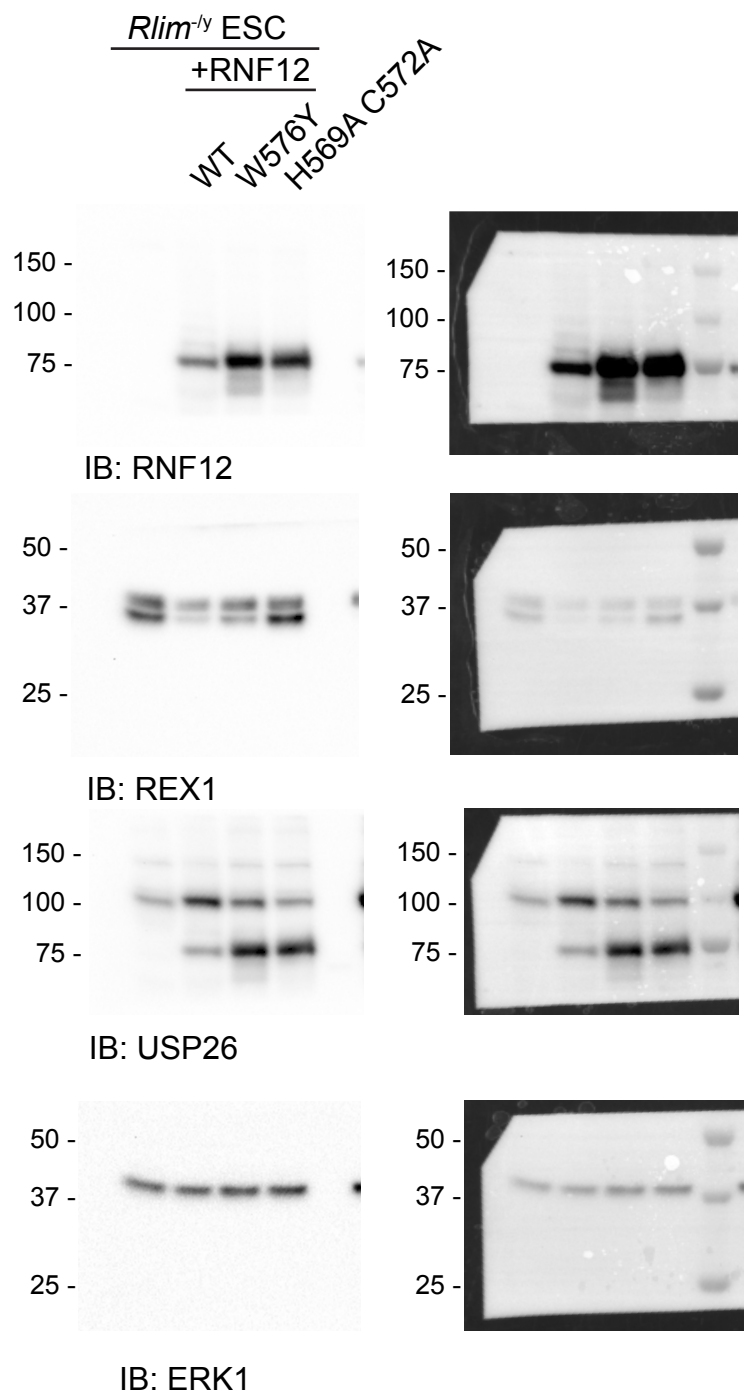

E

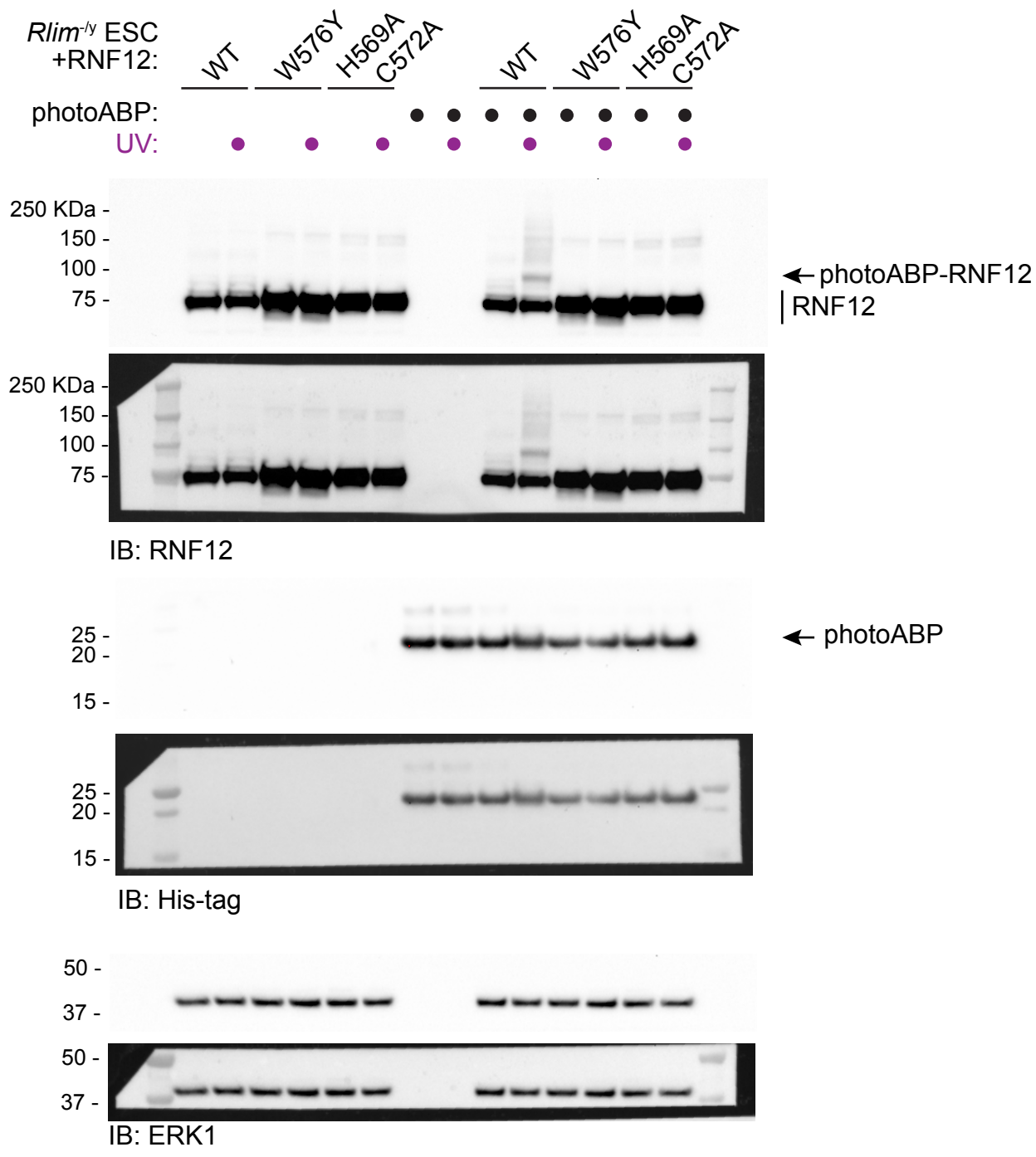

Supplement: Supplementary file 3 [file LSA-2021-01248_SdataF3.pdf]

B

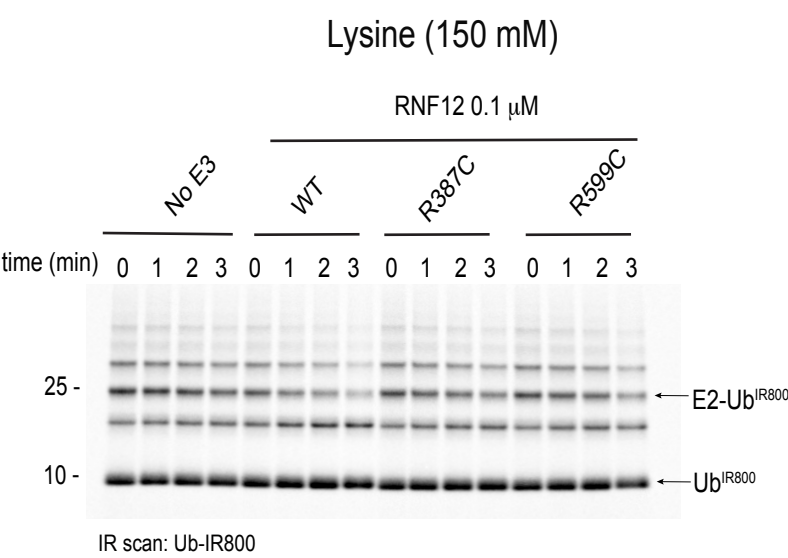

C

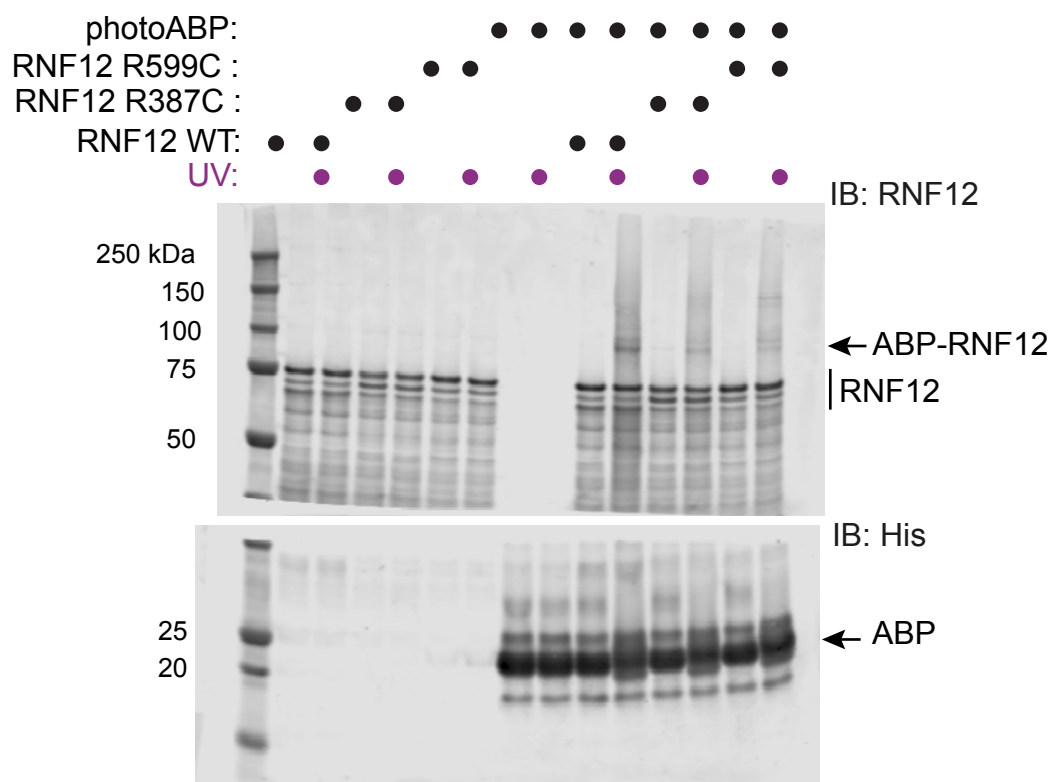

D

*Rlim* ESC:  $\times 1/2$   $1/2$

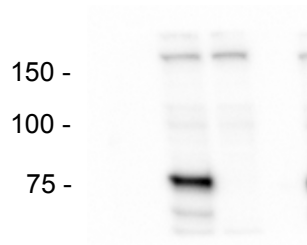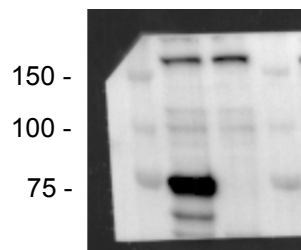

IB: RNF12

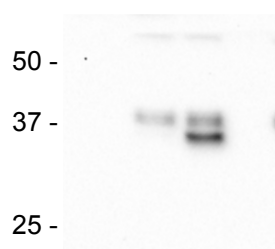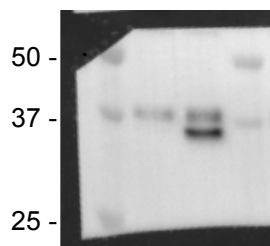

IB: REX1

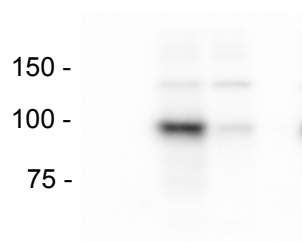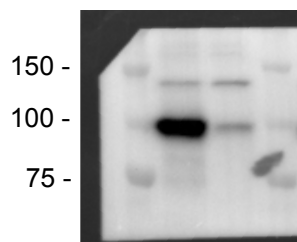

IB: USP26

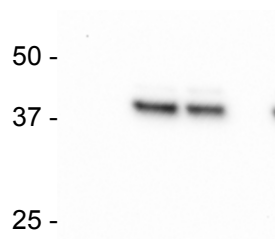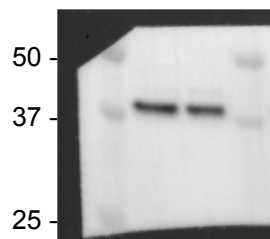

IB: ERK1

E

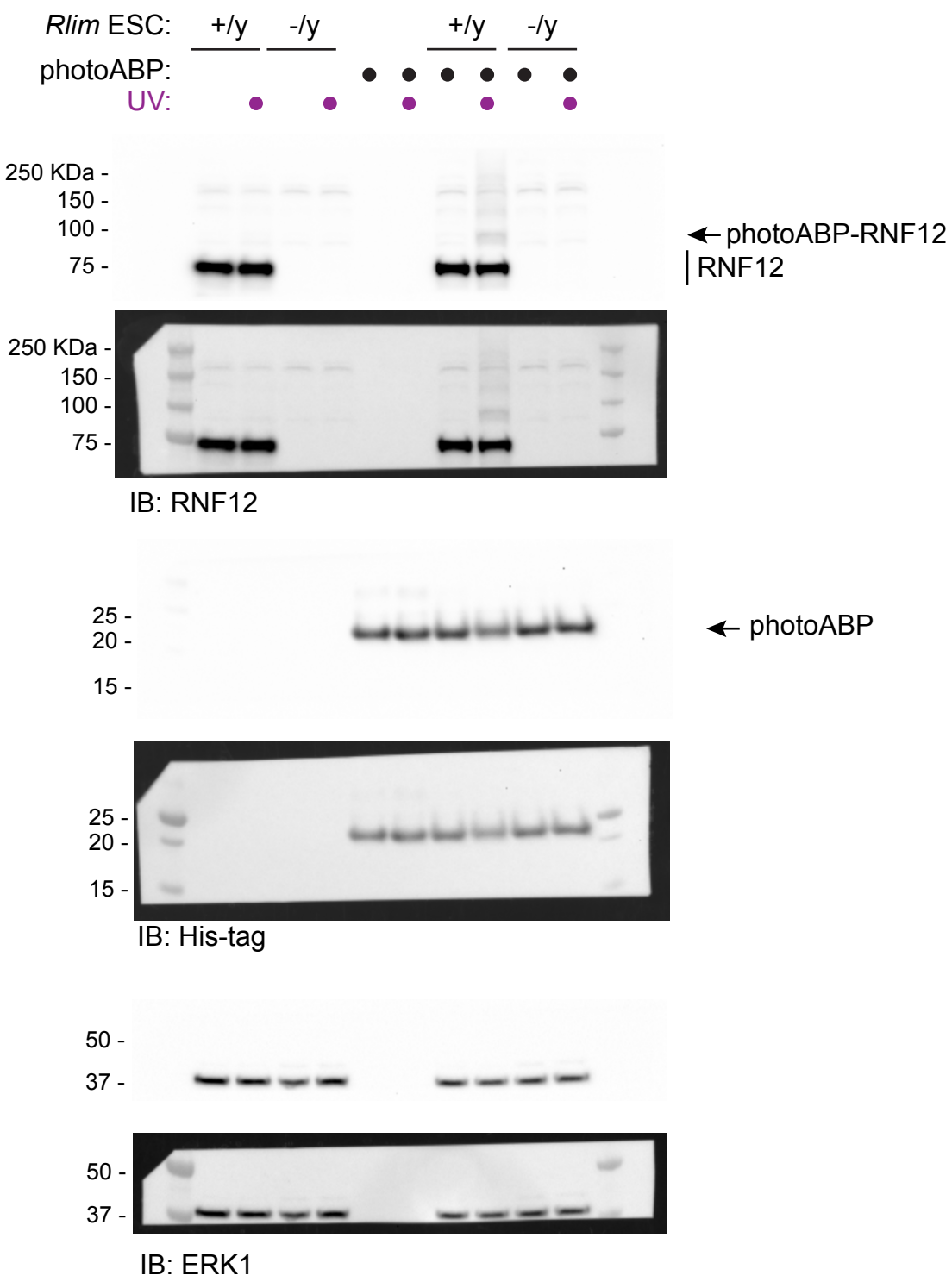

F

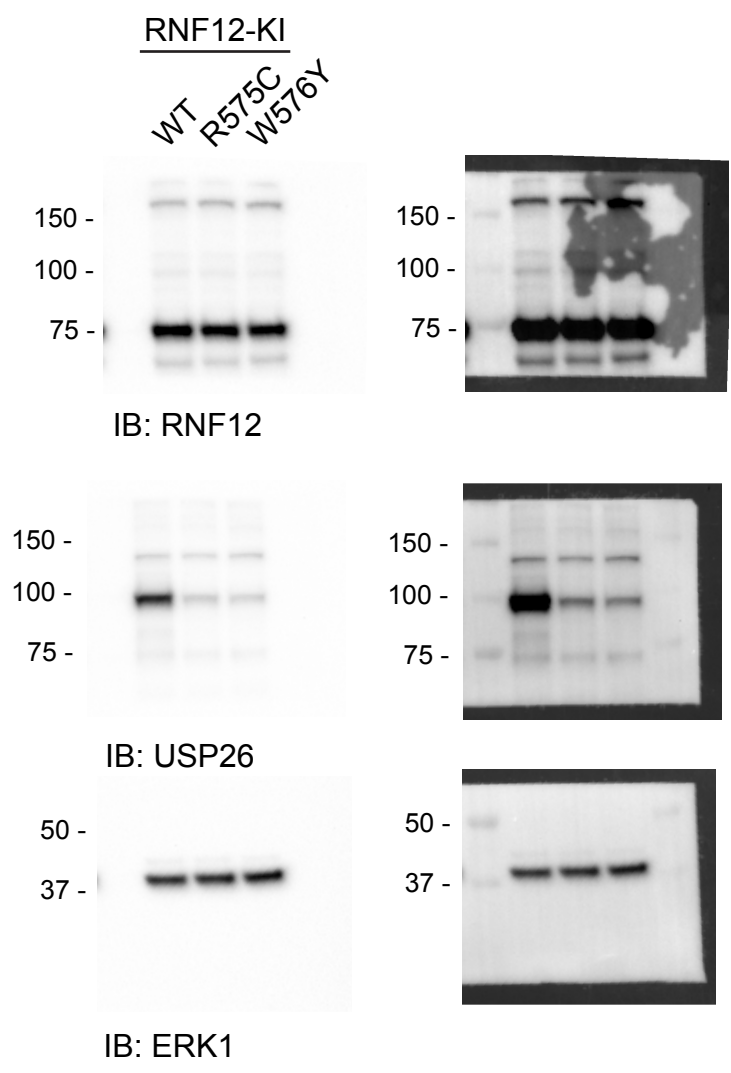

# G

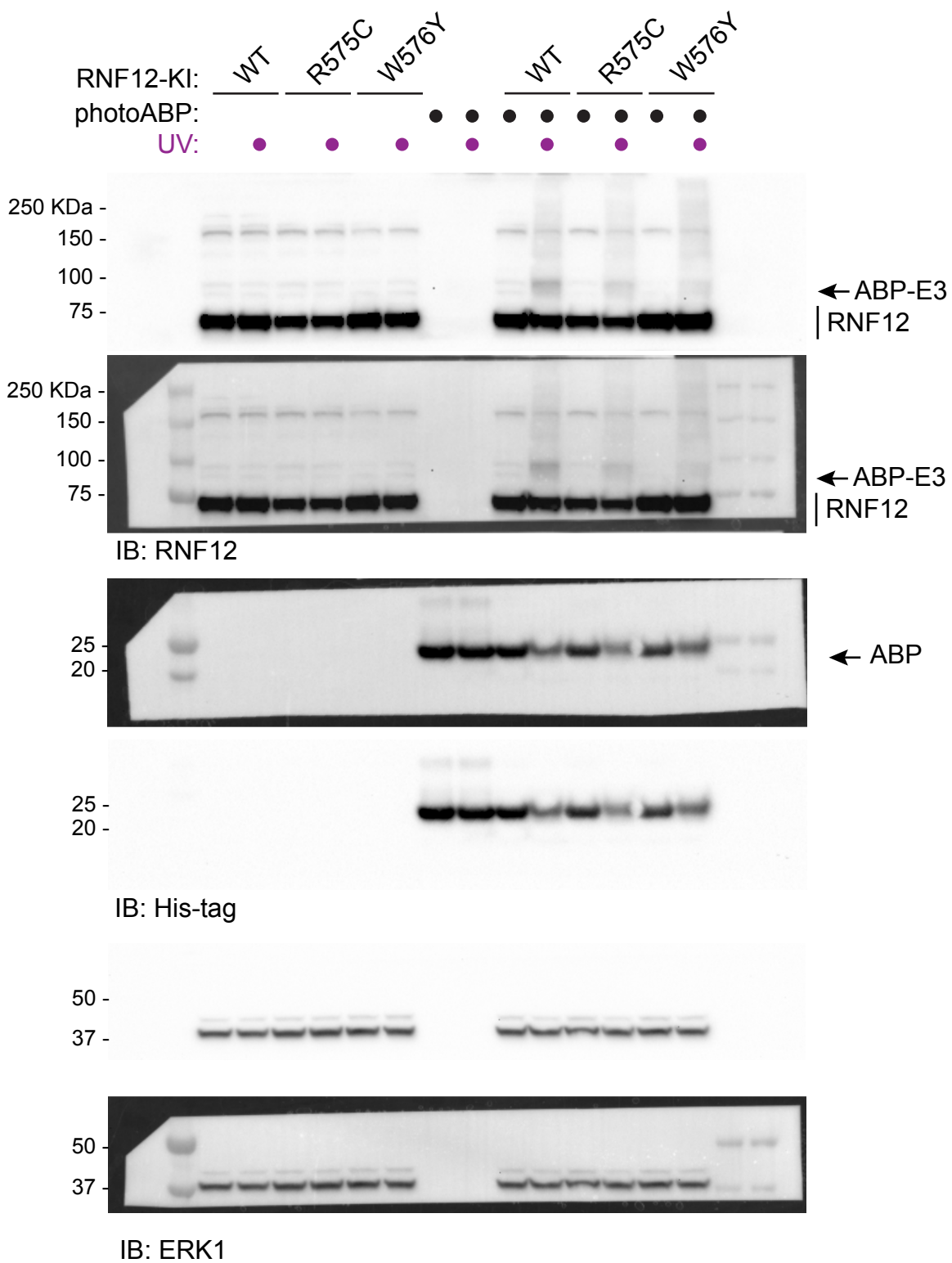

Supplement: Supplementary file 4 [file LSA-2021-01248_SdataF4.pdf]

A

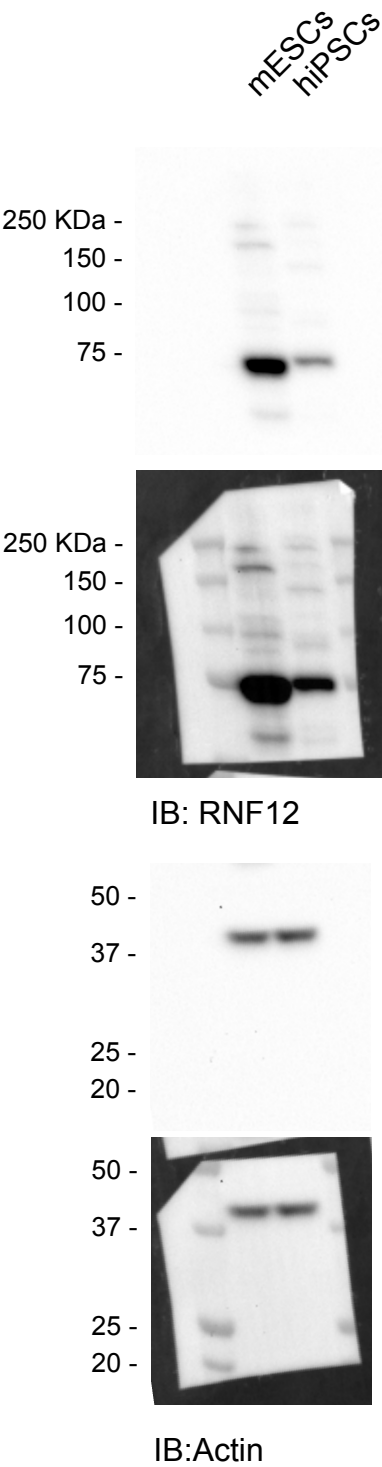

B

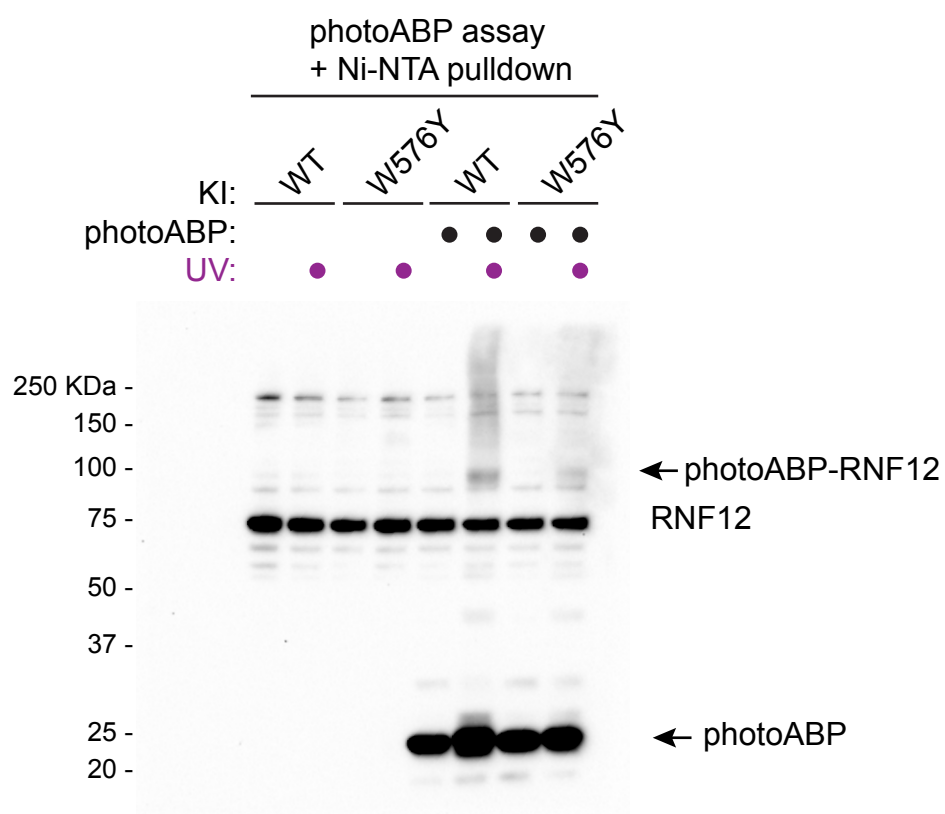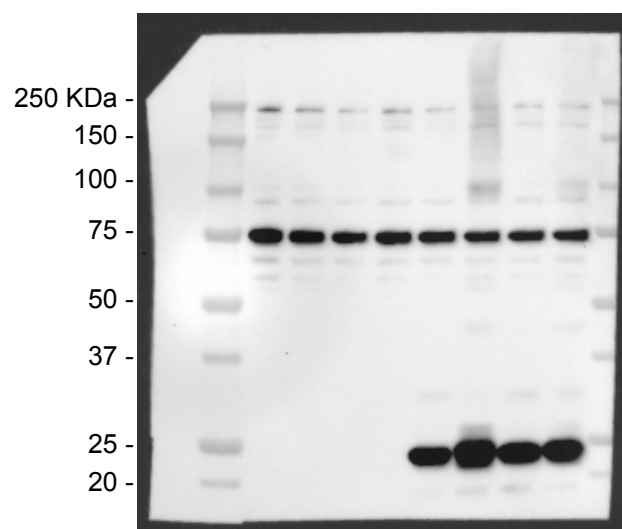

IB: RNF12 / His-tag

C

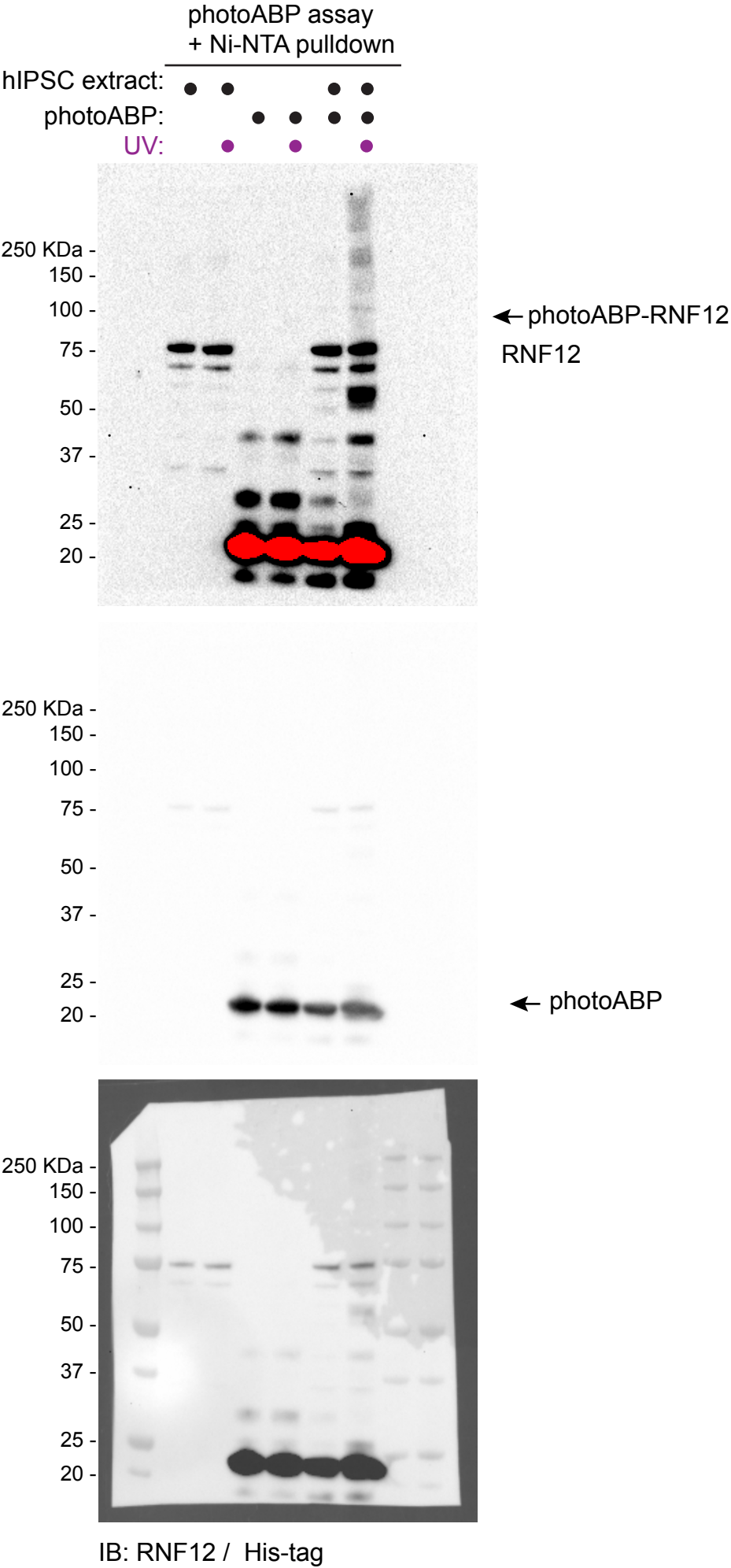

Supplement: Supplementary file 5 [file LSA-2021-01248_SdataF5.pdf]
